# Prognostic Values of Preoperative Inflammatory and Nutritional Markers for Colorectal Cancer

**DOI:** 10.3389/fonc.2020.585083

**Published:** 2020-11-03

**Authors:** Nannan Zhang, Feilong Ning, Rui Guo, Junpeng Pei, Yun Qiao, Jin Fan, Bo Jiang, Yanlong Liu, Zhaocheng Chi, Zubing Mei, Masanobu Abe, Ji Zhu, Rui Zhang, Chundong Zhang

**Affiliations:** ^1^ State key Laboratory of Cancer Biology and National Clinical Research Center for Digestive Diseases, Xijing Hospital of Digestive Diseases, Fourth Military Medical University, Xi’an, China; ^2^ Department of General Surgery, Xuzhou Hospital of Traditional Chinese Medicine, Xuzhou, China; ^3^ Department of Colorectal Surgery, Cancer Hospital of China Medical University, Liaoning Cancer Hospital & Institute, Shenyang, China; ^4^ Department of Gastrointestinal Surgery, The Fourth Affiliated Hospital of China Medical University, Shenyang, China; ^5^ Department of Radiation Oncology, Fudan University Shanghai Cancer Center, Shanghai, China; ^6^ Department of Oncology, Shanghai Medical College of Fudan University, Shanghai, China; ^7^ Department of Colorectal Anal Surgery, Shanxi Province Cancer Hospital & Institute, Taiyuan, China; ^8^ Department of Colorectal Surgery, Harbin Medical University Cancer Hospital, Harbin, China; ^9^ Second Department of Gastrointestinal Surgery, Jilin Cancer Hospital, Changchun, China; ^10^ Department of Anorectal Surgery, Shuguang Hospital, Shanghai University of Traditional Chinese Medicine, Shanghai, China; ^11^ Department of Anorectal Surgery, Anorectal Disease Institute of Shuguang Hospital, Shanghai, China; ^12^ Division for Health Service Promotion, University of Tokyo, Tokyo, Japan; ^13^ Department of Gastrointestinal Surgery, Graduate School of Medicine, University of Tokyo, Tokyo, Japan

**Keywords:** colorectal cancer, inflammatory status, nutritional status, prognostic model, survival outcome

## Abstract

**Background:**

Increasing evidence indicates that inflammation and nutritional status are associated with survival outcomes in patients with colorectal cancer (CRC). This study aimed to investigate the prognostic values of preoperative inflammatory and nutritional factors and develop a prognostic model individually predicting overall survival (OS) and disease-free survival (DFS) in patients with CRC.

**Methods:**

We retrospectively collected data on patients with CRC who underwent radical surgery. Independent prognostic inflammatory and nutritional markers were identified and novel prognostic models were developed incorporating the identified factors. The discriminative ability and model-fitting performance were evaluated by receiver operating characteristic curves and Akaike information criteria. Clinical usefulness was assessed by decision curve analysis.

**Results:**

A total of 400 eligible patients were identified. Multivariate analysis identified pN stage, tumor differentiation grade, neutrophil count, and body mass index as independent prognostic factors for OS, and pN stage, tumor differentiation grade, neutrophil count, neutrophil-lymphocyte ratio, and serum albumin as prognostic factors for DFS. The combined inflammatory and nutritional prognostic model showed better discriminative ability, model-fitting performance, and net benefits than the inflammatory and nutritional models alone, and the American Joint Committee on Cancer (AJCC) 8th TNM classification for predicting OS and DFS.

**Conclusion:**

Preoperative nutritional and inflammatory factors have significant prognostic value in patients with CRC. A novel prognostic model incorporating preoperative inflammatory and nutritional markers provides better prognostic performance than the AJCC 8th TNM classification. A novel nomogram incorporating preoperative inflammatory and nutritional markers can individually predict OS and DFS in patients with CRC.

## Introduction

Colorectal cancer (CRC) is a significant global health burden. It is the third most common cancer in Western countries with more than 140,000 new cases diagnosed in the United States in 2019 and is one of the leading causes of cancer related deaths, with around 700,000 deaths reported globally each year (1, 2). Survival outcomes of advanced CRC have improved as a result of recent advances in surgery, systemic therapy, and best supportive care. The gold standard for predicting survival and surveillance recommendations for CRC remains the Union for International Cancer Control/American Joint Committee on Cancer (UICC/AJCC) tumor/node/metastasis (TNM) anatomical classification (3). However, other factors, including tumor size (4), tumor location (5), tumor deposits (6), lymph node ratio (7), lymphovascular infiltration (8), and carcinoembryonic antigen level (9), have also been associated with patient prognosis and may complement anatomical TNM staging.

Preoperative systemic inflammatory factors play crucial roles in the carcinogenesis and progression of CRC (10) and were proposed as predictors of recurrence and prognostic factors for overall survival (OS) and disease-free survival (DFS) (11). Inflammatory factors, including neutrophil count (12), lymphocyte count (13), neutrophil-lymphocyte ratio (NLR) (14), lymphocyte-monocyte ratio (LMR) (15), and platelet-lymphocyte ratio (PLR) (16) were shown to be independently associated with prognosis in patients with CRC.

Preoperative nutritional factors were also associated with postoperative morbidity and mortality (17, 18), and can be applied as prognostic markers of CRC. Some preoperative nutritional markers, including body mass index (BMI) (19), serum albumin (20), loss of muscle mass (21), and prognostic nutritional index (22) have demonstrated prognostic value in patients with CRC. Thus, some preoperative inflammatory and nutritional factors could predict survival outcomes and might be used to develop a novel prognostic model for patients with CRC.

In this study, we aimed to investigate the prognostic values of preoperative inflammatory and nutritional markers in patients with curable CRC. In addition, we aimed to develop a novel prognostic model incorporating relevant preoperative inflammatory and nutritional markers for the individual prediction of survival outcomes in patients with CRC. We compared the predictive performances of this novel prognostic model with the AJCC 8th TNM classification in terms of model discriminative ability, model-fitting performance, and clinical utility.

## Patients and Methods

### Data Source

We retrospectively collected data on patients with CRC who underwent radical surgery at the Department of Colorectal Surgery, Cancer Hospital of China Medical University, Liaoning Cancer Hospital and Institute. The study was approved by the Ethics Committee of the Cancer Hospital of China Medical University, Liaoning Cancer Hospital and Institute, and written informed consent was obtained from all patients.

### Eligibility Criteria

Patients were included according to the following criteria: (1) pathologically confirmed primary CRC; (2) no other synchronous malignancy; (3) sufficient data regarding the analyzed clinicopathological characteristics; (4) no distant metastasis (M0) before operation; (5) no preoperative treatment (radiotherapy and/or chemotherapy); (6) no history of cancer surgery; (7) pathologically negative resection margins (R0 resection); and (8) postoperative survival ≥ 1 month. Preoperative neoadjuvant therapy may have complex and profound impacts on inflammatory and nutritional factors, and we therefore only explored the prognostic values of preoperative inflammatory and nutritional markers in patients with curable CRC who had not received preoperative neoadjuvant therapy.

### Patient Management

Radical en bloc resection was applied in all patients with curable CRC. All pathological specimens were analyzed independently by two experienced pathologists and disagreements were resolved through discussions. Follow-ups were conducted every 3–6 months for the first 2 years, every 6 months for the next 3 years, and annually thereafter until January 2020 or the time of death. Two doctors were responsible for all follow-ups and for recording all the information. The date of last follow-up was January 2020. OS was defined as the time from the date of surgery to death from any cause, and DFS was defined as the time from surgery to the identification of disease recurrence (23).

### Main Outcomes

The clinical and pathological indexes included history of smoking and drinking, sex, age, operation type, tumor location, tumor size, tumor differentiation grade, number of retrieved lymph nodes, AJCC 8th pathological T stage (pT stage), AJCC 8th pathological N stage (pN stage), vascular invasion, perineural invasion, and adjuvant therapy. Inflammatory and laboratory indexes included white blood cell (WBC) count, neutrophil count, lymphocyte count, platelets, NLR, LWR, PLR, neutrophil-WBC ratio (NWR), and platelet-neutrophil ratio (PNR). The nutritional indexes included serum albumin, hemoglobin, and BMI.

### Statistical Analysis

Categorical data were shown as number (percentage) and continuous data were presented as mean (± standard deviation). Differences in OS and DFS were assessed by Kaplan-Meier survival analysis with log-rank tests. Factors with a *P* value < 0.1 in univariate analyses were considered as potential prognostic factors (24, 25) and were incorporated into Cox proportional hazards regression models, and hazard ratios (HRs) with 95% confidence intervals (CIs) were presented.

The identified independent prognostic factors were applied as basic indexes in the novel prognostic models. We then proposed the following novel prognostic models based on the independent prognostic factors identified by Cox proportional hazards regression models as follows: (1) basic indexes incorporating preoperative inflammatory markers; (2) basic indexes incorporating preoperative nutritional markers; and (3) basic indexes incorporating both preoperative inflammatory and nutritional markers. The AJCC 8th TNM classification was applied as the control model.

The predictive performances of the novel prognostic models were compared in terms of model discriminative ability, model-fitting performance, and clinical utility. Area under the receiver operating characteristic curve (AUC, ROC) analysis was performed to assess model discriminative ability, and Akaike information criteria (AIC) and concordance index (C-index) analyses were carried out to assess model-fitting performance (26). A higher AUC value demonstrated superior model discriminative ability, and a higher C-index or lower AIC value revealed better model-fitting performance. Calibration plots were applied to assess agreements between the predicted and actual probabilities of 3- or 5-year OS and DFS. Decision curve analyses (DCAs) were further applied to measure and compare the clinical utilities of the different prognostic models (27). Novel prognostic nomograms were developed for the individual prediction of survival outcomes.

All statistical analyses were performed using SPSS version 22.0 and R version 3.6.3. All tests were two-sided and a P value < 0.05 was considered statistically significant.

## Results

### Basic Characteristics

A total of 400 patients with CRC were finally included in the study. The clinicopathological characteristics of the included patients are shown in **Table 1**. There were 171 patients with colon cancer and 229 with rectal cancer. The distribution of AJCC 8th TNM classification was 59 patients (14.8%) in stage I, 181 (45.3%) in stage II, and 160 (40.0%) in stage III. The pT stage distribution of the 229 patients with rectal cancer was as follows: 11 pT1 cases, 39 pT2 cases, 117 pT3, and 62 pT4 cases. The median follow-up time was 31 months (range, 1–74 months).

**Table 1:** Clinical, pathological, laboratory and nutritional characteristics.

Variables	Value	Variables	Value
**Clinical factors**		No. of positive LNs	1.80 ( ± 4.61)
Age (years)	60.7 ( ± 11.2)	AJCC 8^th^ pN stage	
Gender		pN0	240 (60.0%)
Male	238 (59.5%)	pN1a	60 (15.0%)
Female	162 (40.5%)	pN1b	41 (10.3%)
History of smoking		pN1c	5 (1.3%)
Yes	93 (23.3%)	pN2a	24 (6.0%)
No	307 (76.8%)	pN2b	30 (7.5%)
History of drinking		AJCC 8^th^ pTNM stage	
Yes	96 (24.0%)	I	59 (14.8%)
No	304 (76.0%)	IIA	127 (31.8%)
Operation type		IIB	45 (11.3%)
Laparoscopic surgery	56 (14.0%)	IIC	9 (2.3%)
Open surgery	344 (86.0%)	IIIA	6 (1.5%)
Adjuvant therapy		IIIB	104 (26.0%)
Yes	219 (54.8%)	IIIC	50 (12.5%)
No	181 (45.3%)	Vascular invasion	
**Pathological factors**		Negative	340 (85.0%)
Tumor location		Positive	60 (15.0%)
Colon	171 (42.8%)	Perineural invasion	
Rectum	229 (57.3%)	Negative	380 (95.0%)
Tumor size (cm)	4.69 ( ± 1.86)	Positive	20 (5.0%)
Tumor differentiation grade		**Inflammatory/laboratory factors**	
Well differentiation	33 (8.3%)	WBC (×10 ^9^/L)	6.61 ( ± 2.07)
Moderate differentiation	326 (81.5%)	Neut (×10^9^/L)	4.13 ( ± 2.12)
Poorly differentiation	37 (9.3%)	Lym (×10^9^/L)	1.87 ( ± 1.71)
Undifferentiation	4 (1.0%)	Plt (×10^9^/L)	268.5 ( ± 89.0)
No. of retrieved LNs (%)	16.2 ( ± 9.64)	NWR	58.7 ( ± 20.1)
Adequate (n ≥ 12)	253 (63.2%)	NLR	2.81 ( ± 4.30)
Inadequate (n < 12)	147 (36.8%)	LWR	27.2 ( ± 9.76)
AJCC 8^th^ pT stage		PNR	109.8 ( ± 175.5)
pT1	17 (4.3%)	PLR	171.4 ( ± 126.7)
pT2	51 (12.8)	**Nutritional factors**	
pT3	212 (53.0%)	Hgb (g/L)	135.3 ( ± 23.2)
pT4a	95 (23.8%)	Alb (g/L)	42.2 ( ± 5.43)
pT4b	25 (6.3%)	BMI (kg/m^2^)	23.5 ( ± 3.31)

AIC, Akaike’s Information Criterion; Alb, albumin; AJCC, American Joint Committee on Cancer; BMI, body mass index; Hgb, hemoglobin; LWR, lymphocyte to white blood cell ratio; Lym, lymphocyte count; LNs, lymph nodes; Neut, neutrophil count; No., number; NLR, neutrophil to lymphocyte ratio; NWR, neutrophil to white blood cell ratio; PLR, platelet to lymphocyte ratio; Plt, platelet count; PNR, platelet to neutrophil ratio; WBC, white blood cell count.

### Independent Prognostic Factors

Univariate analysis identified history of drinking, tumor location, tumor size, tumor differentiation grade, pT stage, pN stage, and adjuvant therapy as potential prognostic factors for OS (log-rank tests, all P < 0.10) (**Table 2**). Tumor differentiation grade (HR 2.12; 95% CI, 1.28–3.53; P = 0.004) and pN stage (HR 1.61; 95% CI, 1.39–1.85; P < 0.001) were further identified as independent prognostic factors for OS by multivariate analysis (**Table 2**).

**Table 2:** Univariate and multivariate analyses of prognostic factors for overall survival.

Variables	No. of patients	Univariate analysis		Multivariate analysis	
		5-year OS	*P* value	HR (95% CI)	*P* value
Age (%)			0.573		
<60 years	184 (46.0)	80.3%			
≥60 years	216 (54.0)	80.7%			
Gender (%)			0.989		
Male	238 (59.5)	79.7%			
Female	162 (40.5)	81.6%			
History of smoking (%)			0.397		
Yes	93 (23.3)	73.7%			
No	307 (76.8)	83.3%			
History of drinking (%)			0.081	1.40 (0.779–2.52)	0.260
Yes	96 (24.0)	71.4%			
No	304 (76.0)	84.3%			
Operation type (%)			0.161		
Laparoscopic surgery	56 (14.0)	92.5%			
Open surgery	344 (86.0)	79.1%			
Adjuvant therapy (%)			0.010	0.889 (0.446–1.77)	0.738
Yes	219 (54.8)	75.3%			
No	181 (45.3)	88.7%			
Tumor location (%)			0.073	1.41 (0.800–2.49)	0.235
Colon	171 (42.8)	75.3%			
Rectum	229 (57.3)	84.5%			
Tumor size (%)			0.063	1.45 (0.810–2.599)	0.211
≤4 cm	182 (45.5)	85.5%			
>4 cm	218 (54.5)	76.9%			
Tumor differentiation grade (%)			<0.001	2.12 (1.28–3.53)	**0.004**
Well differentiation	33 (8.30)	96.8%			
Moderate differentiation	326 (81.5)	81.1%			
Poorly differentiation	37 (9.25)	53.1%			
Undifferentiation	4 (1.00)	66.7%			
No. of retrieved LNs (%)			0.395		
Adequate (n ≥ 12)	253 (63.2)	83.6%			
Inadequate (n < 12)	147 (36.8)	75.2%			
AJCC 8^th^ pT stage (%)			0.005	1.36 (0.965–1.93)	0.078
pT1	17 (4.3)	100%			
pT2	51 (12.8)	90.4%			
pT3	212 (53.0)	81.2%			
pT4a	95 (23.8)	73.4%			
pT4b	25 (6.3)	67.0%			
AJCC 8^th^ pN stage (%)			<0.001	1.61 (1.39–1.85)	**<0.001**
pN0	240 (60.0)	92.7%			
pN1a	60 (15.0)	77.5%			
pN1b	41 (10.3)	63.8%			
pN1c	5 (1.30)	75.0%			
pN2a	24 (6.00)	73.9%			
pN2b	30 (7.50)	26.0%			
Vascular invasion (%)			0.792		
Absence	340 (85.0)	78.9%			
Presence	60 (15.0)	83.4%			
Perineural invasion (%)			0.734		
Absence	380 (95.0)	80.4%			
Presence	20 (5.00)	81.6%			

AJCC, American Joint Committee on Cancer; CI, confidence interval; HR, hazard ratio; LNs, lymph nodes; No., number. Significant values (P < 0.05) are in bold.

Univariate analysis identified a history of drinking, operation type, tumor size, tumor differentiation grade, pT stage, pN stage, and adjuvant therapy as potential prognostic factors for DFS (log-rank tests, all P < 0.10) (**Table 3**), and tumor differentiation grade (HR 2.25; 95% CI, 1.43–3.52; P < 0.001), pT stage (HR 1.52; 95% CI, 1.12–2.06; P = 0.008), and pN stage (HR 1.53; 95% CI, 1.35–1.74; P < 0.001) were further identified as independent prognostic factors for DFS by multivariate analysis (**Table 3**). These identified prognostic factors were applied as basic indexes in the novel prognostic models.

**Table 3:** Univariate and multivariate analyses of prognostic factors for disease-free survival.

Variables	No. of patients	Univariate analysis		Multivariate analysis	
		5-year DFS	*P* value	HR (95% CI)	*P* value
Age (%)			0.665		
<60 years	184 (46.0)	77.8%			
≥60 years	216 (54.0)	78.1%			
Gender (%)			0.598		
Male	238 (59.5)	76.0%			
Female	162 (40.5)	80.9%			
History of smoking (%)			0.375		
Yes	93 (23.3)	70.1%			
No	307 (76.8)	81.1%			
History of drinking (%)			0.097	1.32 (0.776–2.24)	0.307
Yes	96 (24.0)	67.9%			
No	304 (76.0)	82.1%			
Operation type (%)			0.049	0.661 (0.194–2.26)	0.508
Laparoscopic surgery	56 (14.0)	92.5%			
Open surgery	344 (86.0)	76.1%			
Adjuvant therapy (%)			0.002	0.843 (0.443–1.60)	0.603
Yes	219 (54.8)	71.9%			
No	181 (45.3)	86.6%			
Tumor location (%)			0.116		
Colon	171 (42.8)	75.5%			
Rectum	229 (57.3)	82.3%			
Tumor size (%)			0.069	1.31 (0.779–2.21)	0.309
≤4 cm	182 (45.5)	82.9%			
>4 cm	218 (54.5)	74.4%			
Tumor differentiation grade (%)			<0.001	2.25 (1.43–3.52)	**<0.001**
Well differentiation	33 (8.30)	96.8%			
Moderate differentiation	326 (81.5)	78.6%			
Poorly differentiation	37 (9.25)	48.8%			
Undifferentiation	4 (1.00)	66.7%			
No. of retrieved LNs (%)			0.958		
Adequate (n ≥ 12)	253 (63.2)	74.6%			
Inadequate (n < 12)	147 (36.8)	80.0%			
AJCC 8^th^ pT stage (%)			<0.001	1.52 (1.12–2.06)	**0.008**
pT1	17 (4.3)	100%			
pT2	51 (12.8)	90.4%			
pT3	212 (53.0)	78.8%			
pT4a	95 (23.8)	71.5%			
pT4b	25 (6.3)	54.2%			
AJCC 8^th^ pN stage (%)			<0.001	1.53 (1.35–1.74)	**<0.001**
pN0	240 (60.0)	90.4%			
pN1a	60 (15.0)	77.3%			
pN1b	41 (10.3)	62.3%			
pN1c	5 (1.30)	75.0%			
pN2a	24 (6.00)	61.0%			
pN2b	30 (7.50)	22.6%			
Vascular invasion (%)			0.832		
Absence	340 (85.0)	76.5%			
Presence	60 (15.0)	80.3%			
Perineural invasion (%)			0.623		
Absence	380 (95.0)	78.0%			
Presence	20 (5.00)	77.5%			

AJCC, American Joint Committee on Cancer; CI, confidence interval; DFS, disease-free survival; HR, hazard ratio; LNs, lymph nodes; No., number. Significant values (P < 0.05) are in bold.

### Preoperative Inflammatory and Nutritional Markers

The preoperative inflammatory markers of WBC count, neutrophil count, NWR, LWR, and PNR were identified as potential predictive factors for OS, and WBC count, neutrophil count, NWR, NLR, LWR, PNR, and PLR as potential predictive factors for DFS by univariate analyses (log-rank tests, all P < 0.10) (**Table 4**). The preoperative nutritional markers hemoglobin, serum albumin, and BMI were potential predictive factors for both OS and DFS (log-rank tests, all P < 0.10) (**Table 4**).

**Table 4:** Univariate analysis of preoperative inflammatory and nutritional factors.

Variables	Overall survival		Disease-free survival	
	HR (95% CI)	*P* value	HR (95% CI)	*P* value
**Inflammatory/laboratory data**				
WBC (×10^9^/L)	1.11 (1.01–1.23)	**0.035**	1.11 (1.01–1.21)	**0.025**
Neut (×10^9^/L)	1.15 (1.06–1.24)	**<0.001**	1.14 (1.06–1.22)	**0.001**
Lym (×10^9^/L)	0.817 (0.524–1.28)	0.375	0.723 (0.471–1.11)	0.139
Plt (×10^9^/L)	1.00 (0.997–1.00)	0.991	1.00 (0.997–1.00)	0.849
NWR	1.02 (1.00–1.04)	**0.026**	1.02 (1.00–1.04)	**0.024**
NLR	1.01 (0.974–1.05)	0.564	0.960 (0.963–0.986)	**0.003**
LWR	0.961 (0.933–0.989)	**0.007**	1.06 (1.04–1.09)	**<0.001**
PNR	0.988 (0.977–0.998)	**0.024**	0.996 (0.992–1.00)	**0.088**
PLR	1.00 (0.998–1.00)	0.995	1.002 (1.001–1.003)	**<0.001**
**Nutritional data**				
Hgb (g/L)	0.602 (0.331–1.10)	**0.096**	0.634 (0.368–1.09)	**0.100**
Alb (g/L)	0.405 (0.224–0.730)	**0.003**	0.444 (0.259–0.759)	**0.003**
BMI (kg/m^2^)	0.328 (0.178–0.605)	**<0.001**	0.450 (0.249–0.814)	**0.008**

Alb, albumin; BMI, body mass index; CI, confidence interval; Hgb, hemoglobin; HR, hazard ratio; LWR, lymphocyte to white blood cell ratio; Lym, lymphocyte count; Neut, neutrophil count; NLR, neutrophil to lymphocyte ratio; NWR, neutrophil to white blood cell ratio; PLR, platelet to lymphocyte ratio; Plt, platelet count; PNR, platelet to neutrophil ratio; WBC, white blood cell count; Hgb, ≤120 vs. >120 g/L; Alb, ≤38 vs. >38 g/L; BMI, ≤19.5 vs. >19.5 kg/m^2^. Significant values (P < 0.05) are in bold.

### Development of Novel Prognostic Models

We developed an inflammatory model predicting OS including tumor differentiation grade, pN, stage and neutrophil count, and an inflammatory model predicting DFS including pT stage, pN stage, tumor differentiation grade, neutrophil count, and NLR (**Tables 5** and **6**). We also developed a nutritional model predicting OS including pN stage, tumor differentiation grade, and BMI, and a nutritional model predicting DFS including pN stage, tumor differentiation grade, and serum albumin (**Tables 5** and **6**). The combined inflammatory and nutritional model predicting OS included pN stage, tumor differentiation grade, neutrophil count, and BMI, and the equivalent model predicting DFS included pN stage, tumor differentiation grade, neutrophil count, NLR, and serum albumin (**Tables 5** and **6**).

**Table 5:** Prognostic performances of different models in terms of overall survival.

Prognostic models	Multivariate analysis		AUC (95% CI)	C-index	AIC
	HR (95% CI)	*P* value			
**Inflammatory/laboratory model**			0.796 (0.720–0.872)	0.794	522.6
pN stage	1.62 (1.42–1.851)	**<0.001**			
Tumor differentiation grade	2.56 (1.58–4.15)	**<0.001**			
pT stage^#^		0.051			
WBC (×10^9^/L)		0.299			
Neut (×10^9^/L)	1.19 (1.09–1.31)	**<0.001**			
NWR		0.863			
LWR		0.960			
PNR		0.178			
**Nutritional model**			0.793 (0.722–0.864)	0.782	521.5
pN stage	1.60 (1.40–1.83)	**<0.001**			
Tumor differentiation grade	2.84 (1.73–4.67)	**<0.001**			
pT stage^#^		0.181			
Hgb (g/L)		0.132			
Alb (g/L)		0.095			
BMI (kg/m^2^)	0.329 (0.177–0.613)	**<0.001**			
**Inflammatory and nutritional model**			0.820 (0.749–0.890)	0.813	513.6
pN stage	1.60 (1.40–1.83)	**<0.001**			
Tumor differentiation grade	2.80 (1.72–4.57)	**<0.001**			
pT stage^#^		0.161			
WBC (×10^9^/L)		0.466			
Neut (×10^9^/L)	1.21 (1.10–1.33)	**<0.001**			
NWR		0.957			
LWR		0.603			
PNR		0.241			
Hgb (g/L)		0.106			
Alb (g/L)		0.283			
BMI (kg/m^2^)	0.311 (0.166–0.582)	**<0.001**			
**Control model**			0.789 (0.716–0.862)	0.776	535.3
AJCC 8^th^ pTNM (pT, pN)					

AIC, Akaike’s Information Criterion; Alb, albumin; AJCC, American Joint Committee on Cancer; BMI, body mass index;

CI, confidence interval; C-index, concordance index; Hgb, hemoglobin; HR, hazard ratio; LWR, lymphocyte to white blood cell ratio; NWR, neutrophil to white blood cell ratio; PNR, platelet to neutrophil ratio; WBC, white blood cell count;

^#^pT stage was not significant in univariate analysis but still included in the multivariate analysis;

Hgb, ≤120 vs. >120 g/L; Alb, ≤38 vs. >38 g/L; BMI, ≤19.5 vs. >19.5 kg/m^2^. Significant values (P < 0.05) are in bold.

**Table 6:** Prognostic performances of different models in terms of disease-free survival.

Prognostic models	Multivariate analysis		AUC (95% CI)	C-index	AIC
	HR (95% CI)	*P* value			
**Inflammatory/laboratory model**			0.784 (0.715–0.854)	0.785	646.6
pT stage	1.40 (1.03–1.91)	**0.031**			
pN stage	1.55 (1.37–1.76)	**<0.001**			
Tumor differentiation grade	1.91 (1.21–3.02)	**0.005**			
WBC (×10^9^/L)		0.545			
Neut (×10^9^/L)	1.11 (1.01–1.22)	**0.032**			
NWR		0.656			
LWR		0.820			
NLR	1.04 (1.01–1.08)	**0.014**			
PNR		0.196			
PLR		0.835			
**Nutritional model**			0.779 (0.714–0.846)	0.767	653.3
pT stage		0.059			
pN stage	1.60 (1.42–1.80)	**<0.001**			
Tumor differentiation grade	2.12 (1.37–3.29)	**0.001**			
Hgb (g/L)		0.433			
Alb (g/L)	0.454 (0.263–0.785)	**0.005**			
BMI (kg/m^2^)		0.061			
**Inflammatory and nutritional model**			0.803 (0.738–0.869)	0.777	648.0
pT stage		0.066			
pN stage	1.59 (1.41–1.79)	**<0.001**			
Tumor differentiation grade	2.15 (1.39–3.33)	**0.001**			
WBC (×10^9^/L)		0.660			
Neut (×10^9^/L)	1.11 (1.01–1.22)	**0.039**			
NWR		0.670			
LWR		0.985			
NLR	1.04 (1.02–1.08)	**0.014**			
PNR		0.232			
PLR		0.745			
Hgb (g/L)		0.579			
Alb (g/L)	0.522 (0.293–0.926)	**0.027**			
BMI (kg/m^2^)		0.055			
**Control model**			0.784 (0.718–0.850)	0.764	668.4
AJCC 8^th^ pTNM (pT, pN)					

AJCC, American Joint Committee on Cancer; AIC, Akaike’s Information Criterion; Alb, albumin; BMI, body mass index;

CI, confidence interval; C-index, concordance index; Hgb, hemoglobin; HR, hazard ratio; LWR, lymphocyte to white blood cell ratio;

NLR, neutrophil to lymphocyte ratio; NWR, neutrophil to white blood cell ratio; PLR, platelet to lymphocyte ratio;

PNR, platelet to neutrophil ratio; WBC, white blood cell count;

Hgb, ≤120 vs. >120 g/L; Alb, ≤38 vs. >38 g/L; BMI, ≤19.5 vs. >19.5 kg/m^2^. Significant values (P< 0.05) are in bold.

### Assessment of Predictive Performance

**Figure 1:**
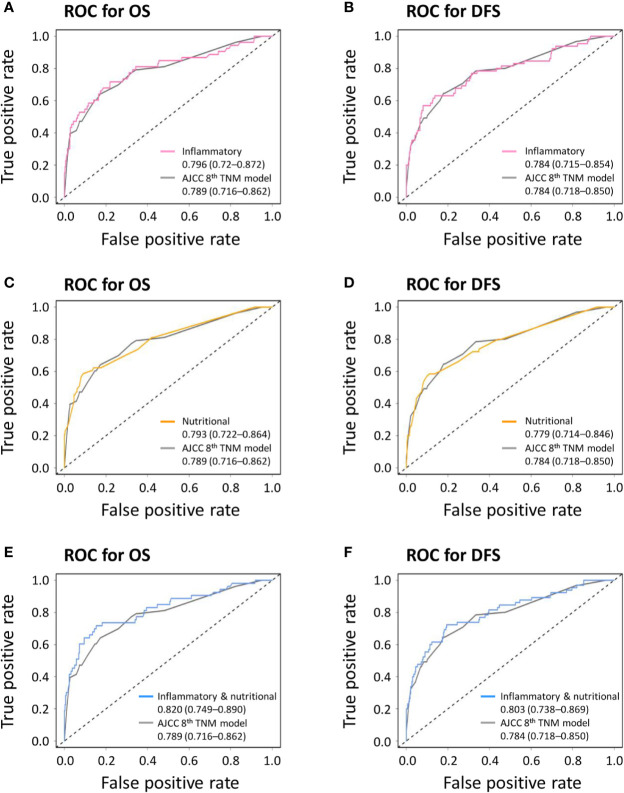
Receiver operating characteristic curves (ROC) comparing discriminative abilities of novel prognostic models with the American Joint Committee on Cancer (AJCC) 8th TNM classification. **(A)** Inflammatory model versus AJCC 8th TNM classification for overall survival (OS); **(B)** inflammatory model versus AJCC 8th TNM classification for disease-free survival (DFS); **(C)** nutritional model versus AJCC 8th TNM classification for OS; **(D)** nutritional model versus AJCC 8th TNM classification for DFS; **(E)** combined inflammatory and nutritional model versus AJCC 8th TNM classification for OS; **(F)** combined inflammatory and nutritional model versus AJCC 8th TNM classification for DFS.

The combined inflammatory and nutritional model showed superior model discriminative ability in terms of OS (AUC, 0.820; 95% CI, 0.749–0.890) compared with the inflammatory model (AUC, 0.796; 95% CI, 0.72–0.872), nutritional model (AUC, 0.793; 95% CI, 0.722–0.864), and the AJCC 8th TNM classification (AUC, 0.789; 95% CI, 0.716–0.862) (**Figure 1** and **Table 2**). It also showed better model-fitting performance (C-index, 0.813; AIC, 513.6) than the inflammatory model (C-index, 0.794; AIC, 522.6), nutritional model (C-index, 0.782; AIC, 521.5), and AJCC 8th TNM classification (C-index, 0.776; AIC, 535.3) (**Table 5**).

**Figure 2:**
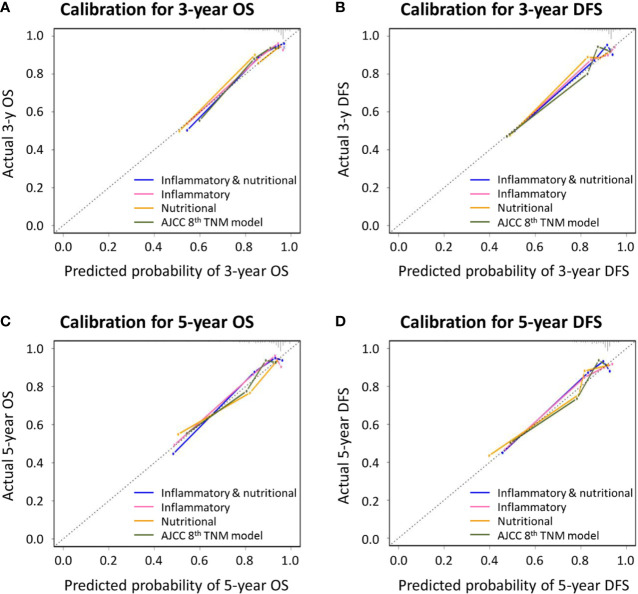
Calibration curves for novel prognostic models and the American Joint Committee on Cancer (AJCC) 8th TNM classification for predicting **(A)** 3-year overall survival (OS); **(B)** 3-year disease-free survival (DFS); **(C)** 5-year OS; and **(D)** 5-year DFS.

Similar comparative results were found in terms of model-fitting performance and model discriminative ability for DFS (**Table 6** and **Figure 1**). Furthermore, the calibration plots of all models showed good agreement between the predicted and actual probabilities of 3- or 5-year OS and DFS (**Figure 2**).

### Clinical Utility Estimated by DCA

**Figure 3:**
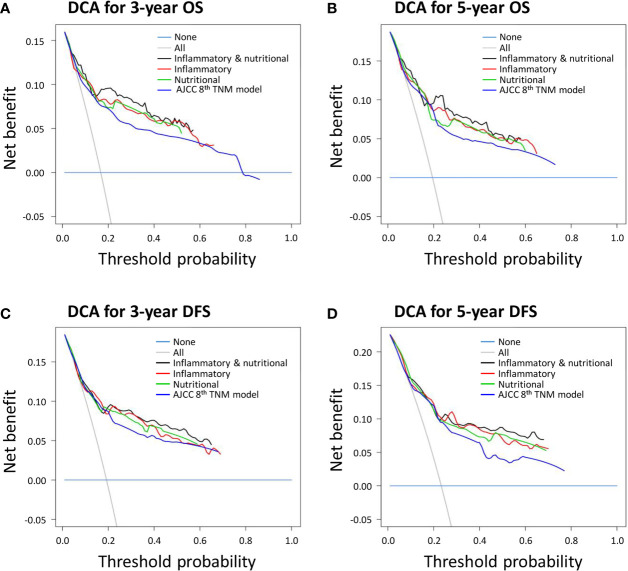
Decision curve analyses (DCAs) of novel prognostic models and the American Joint Committee on Cancer (AJCC) 8th TNM classification for **(A)** 3-year overall survival (OS); **(B)** 3-year disease-free survival (DFS); **(C)** 5-year OS; and **(D)** 5-year DFS.

DCA was performed to estimate the clinical utilities of the novel prognostic models and the control AJCC 8th TNM classification. The combined inflammatory and nutritional model revealed superior net benefits over the inflammatory and nutritional models alone in terms of predicting 3- and 5-year OS between the threshold probabilities of 15%–45% and 20%–55%, respectively (**Figures 3A, B**). All three novel prognostic models showed superior net benefits compared with the AJCC 8th TNM classification for predicting 3- and 5-year OS between the threshold probabilities of 15%–60% and 20%–60%, respectively. Similar findings were obtained for DCAs predicting 3- and 5-year DFS (**Figures 3C, D**).

### Nomograms Individually Predicting Survival

**Figure 4:**
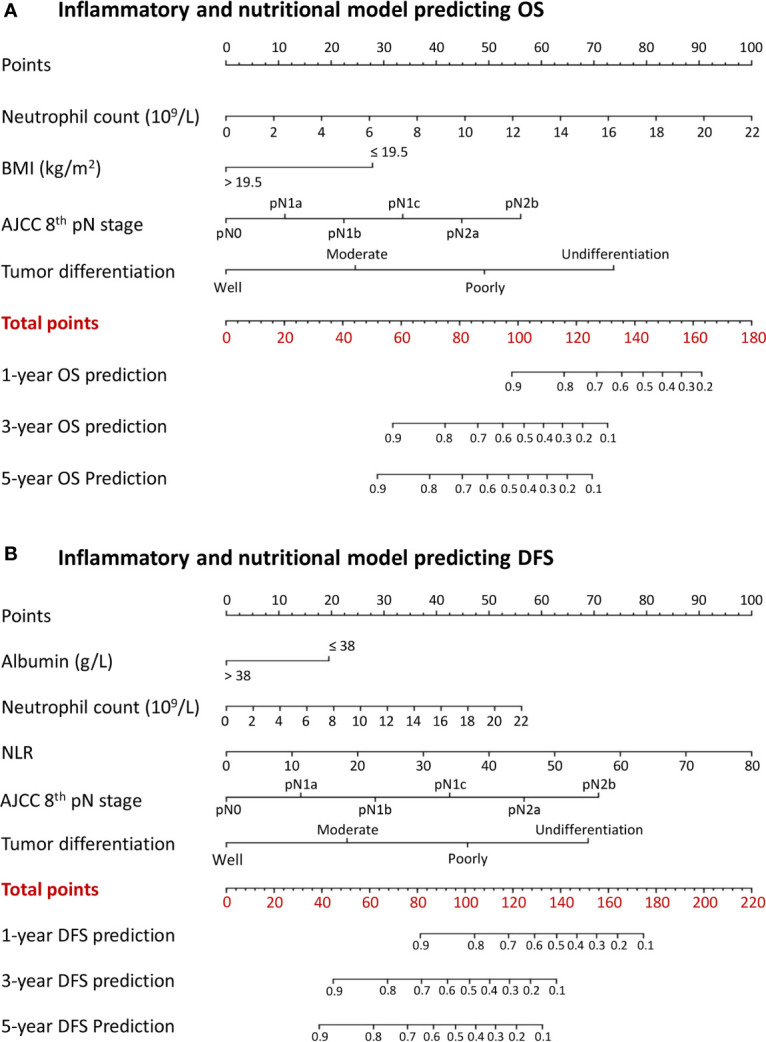
Nomograms conveying the results of the novel prognostic models incorporating clinicopathological characteristics and preoperative inflammatory and nutritional factors for predicting **(A)** overall survival (OS) and **(B)** disease-free survival (DFS) in patients with colorectal cancer. BMI, body mass index; NLR, neutrophil-lymphocyte ratio.

**Figure 5:**
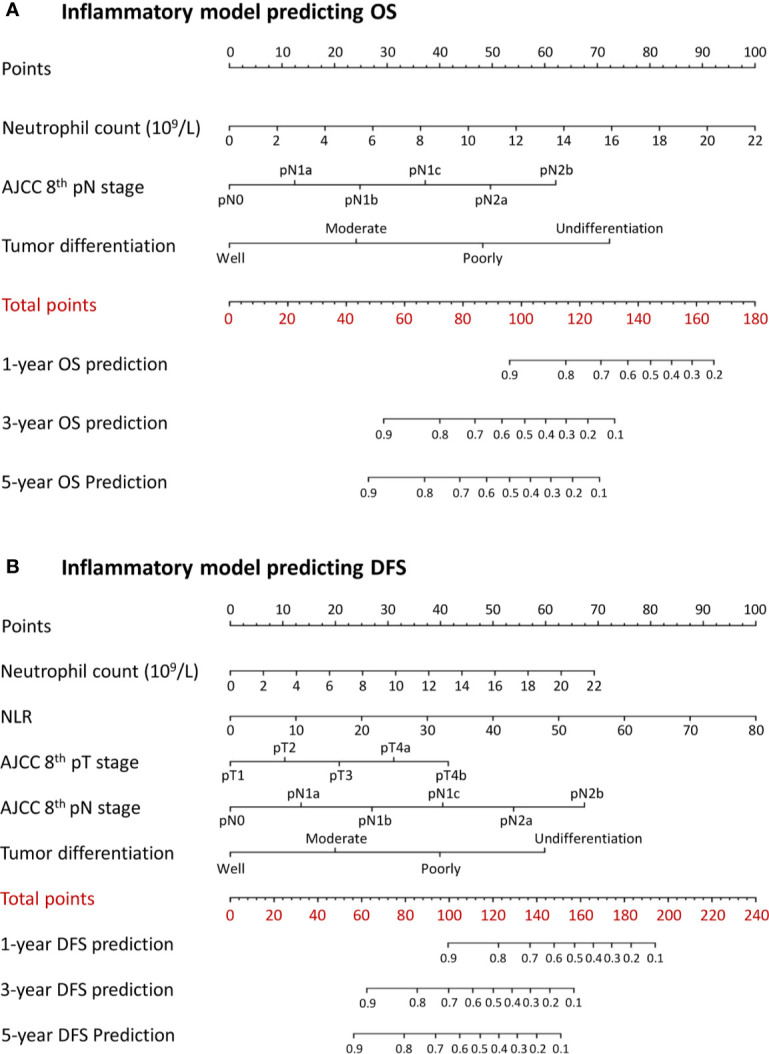
Nomograms conveying the results of the novel prognostic models incorporating clinicopathological characteristics and preoperative inflammatory factors for predicting **(A)** overall survival (OS) and **(B)** disease-free survival (DFS) in patients with colorectal cancer. NLR, neutrophil-lymphocyte ratio.

**Figure 6:**
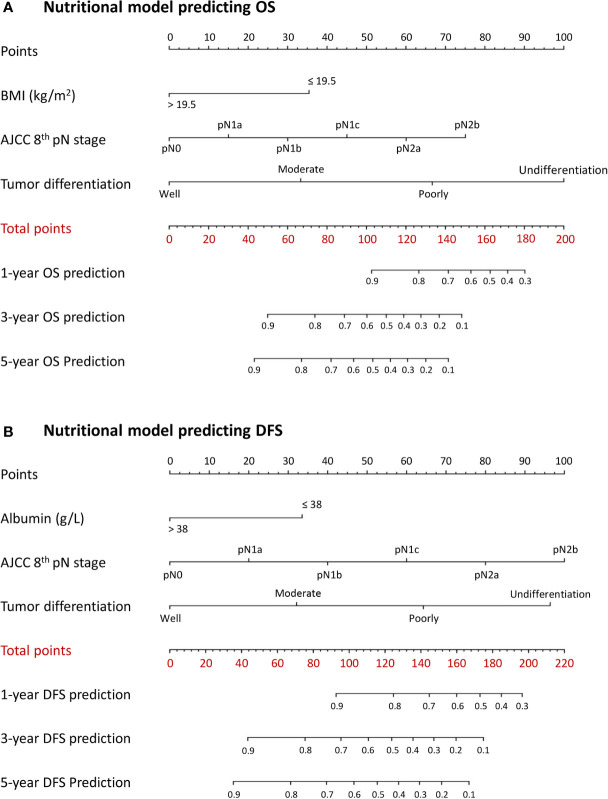
Nomograms conveying the results of the novel prognostic models incorporating clinicopathological characteristics and preoperative nutritional factors for predicting **(A)** overall survival (OS) and **(B)** disease-free survival (DFS) in patients with colorectal cancer. BMI, body mass index; NLR, neutrophil-lymphocyte ratio.

A combined inflammatory and nutritional nomogram including pN stage, tumor differentiation grade, neutrophil count, and BMI was established for individually predicting 1-, 3-, and 5-year OS (**Figure 4A**), and a combined nomogram including pN stage, tumor differentiation grade, neutrophil count, NLR, and serum albumin was established for individually predicting 1-, 3-, and 5-year DFS (**Figure 4B**). Inflammatory and nutritional nomograms were also established (**Figures 5** and **6**).

## Discussion

Increasing evidence has demonstrated decisive roles for inflammatory and nutritional indexes in the prognosis of cancers, including CRC (10, 17, 18). Although the AJCC TNM anatomical classifications have been regarded as the most robust prognostic indicator for CRC (3), systemic inflammation and nutritional markers can provide additional prognostic value to complement this classification (3). We therefore investigated the associations between preoperative systemic inflammation and nutritional statuses and survival outcomes in patients with CRC. We further developed novel prognostic models based on identified independent nutritional and inflammatory prognostic factors for individually predicting OS and DFS in patients with CRC undergoing curative surgery.

Systemic inflammation caused by complicated host-tumor interactions is an important component of tumors and plays a pivotal role in cancer initiation, development, and metastasis (28, 29). Many inflammatory parameters have been reported to be independently associated with the prognosis of CRC, including neutrophil count (12), lymphocyte count (13), NLR (14), LMR (15), and PLR (16). We thus collected and analyzed information on markers previously documented to be related to the prognosis of CRC. The current findings demonstrated that neutrophil count was an independent predictor of OS. Neutrophils, as a hallmark of cancer, reflect the status of host inflammation and participate in different stages of the oncogenic processes of tumor initiation, growth, proliferation, and metastatic spread (30, 31). Tumor initiation can be promoted by the production of matrix metalloproteinase 9 and the release of reactive oxygen species, reactive nitrogen species, or proteases by neutrophils (32, 33). Neutrophils can also mediate and facilitate tumor proliferation by attenuating the immune system and *via* degradation of insulin receptor substrate 1 and the activation of phosphoinositide 3-kinase signaling as a result of the transfer of neutrophil elastase to cancer cells (34, 35).

The current study indicated that neutrophils and the NLR were also independent predictors of DFS, and we hypothesized that they might be associated with tumor recurrence in patients with CRC. Neutrophils can facilitate metastatic spread by inhibiting natural killer function and promoting the extravasation of tumor cells (36). An elevated NLR indicates a systemic inflammatory status, which, in turn, suggests neutrophilia, lymphocytopenia, or a combination of both. The tumor phenotype can also promote the influx of inflammatory lymphocytes around the tumor leading to cell destruction within the surrounding tissues, thereby generating a broader nonspecific inflammatory response (37). In addition, lymphocytopenia may lead to a poorer lymphocyte-mediated immune response to malignancy, and a subsequently increased potential for tumor recurrence (38).

Previous studies showed an association between preoperative nutritional status and mortality in patients with gastrointestinal cancers (17, 18). Here, we attempted to apply objective and easily measurable markers of nutritional status, and found that underweight patients had significantly poorer OS and BMI was identified as an independent predictive factor of OS. These findings were consistent with previous studies (39, 40), which showed that BMI was associated with prognosis (40), and a high metabolic rate and anorexia induced by cancer may lead to patients being underweight (39). Furthermore, cytokine responses and subsequent immune system activation were impaired in patients with chronic malnutrition and micronutrient deficiency, potentially influencing interactions between the tumor and immune system (41). Tumor cells have been shown to interact with circulating immune cells *via* various molecular signals, from initial carcinogenesis to metastasis (42).

Serum albumin was identified as an independent predictor of DFS in patients with CRC in the current study. Although serum albumin cannot comprehensively reflect the patient’s nutritional status, it has nevertheless been used extensively and is accepted as a good indicator of nutritional status (43). Serum albumin was also reported to play a potentially important protective role in promoting the removal of reactive oxygen species, which is a process related to the pathogenesis of many diseases, including cancers (44). Cancer-related systemic inflammation, malnutrition, and an increased turnover of albumin by tumors can result in the inhibition of albumin synthesis and a reduction in serum albumin (45). The relationship between serum albumin and survival might also be influenced by an elevation in cytokines (46). C-reactive protein, tumor necrosis factor, and interleukin-1 are involved in the pathogenesis of cancer, and can decrease serum albumin concentrations (46, 47). Alternatively, tumor necrosis factor may increase the permeability of the microvasculature, thus allowing the increased transcapillary passage of albumin and leading to low levels of serum albumin. Serum interleukin-6 is also increased in acute and chronic inflammatory situations and has been shown to be associated with hypoalbuminemia (48).

Nutritional status can also be a marker of systemic inflammatory responses, and some studies have demonstrated a relationship between acute-phase reactants and survival in patients with cancer. They indicated that hypoalbuminemia was a marker of malnutrition or systemic inflammation, because some proinflammatory substances, such as cytokines, reduced the concentration of albumin (49, 50). Furthermore, significant inflammation or injury can influence appetite, gastrointestinal motility, and hemodynamic stability, which may, in turn, affect the nutritional status of patients (51).

Many prognostic models incorporating different factors have been developed to predict the prognosis or risk of death in patients with CRC. Bibault et al. developed and validated a model incorporating tumor features and patient medical and demographic information to predict survival in CRC, with a high predictive performance (AUC, 0.84; accuracy, 0.83) at the individual scale (52). Dienstmann et al. confirmed the importance of genomic markers, transcriptomic subtyping, and microenvironmental features for survival prediction in patients with stage II/III CRC (53, 54). Importantly, establishment of the nomogram improved the individual prediction of survival outcomes for patients with CRC (55, 56).

We developed several novel prognostic models incorporating the nutritional and inflammatory prognostic factors identified in this study. The combined inflammatory and nutritional prognostic model showed superior model discriminative ability, better model-fitting performance, and higher net benefits compared with either the inflammatory or nutritional model alone. The AJCC 8th TNM classification is considered to be the gold standard for prognostic prediction in patients with CRC. However, the present study demonstrated that the novel combined inflammatory and nutritional prognostic model also had superior predictive performances for OS and DFS compared with the AJCC 8th TNM classification. A nomogram is a visible tool for individually predicting survival outcomes, with high predictive accuracy and comprehensive outcomes in cancer (57). Nomograms may help clinicians to predict individual survival outcomes for patients with CRC after curative surgery, and may provide useful information in terms of recommending postoperative adjuvant therapy or intensive follow-up. Importantly, the markers required for this novel prognostic nomogram model could be easily obtained, making it a convenient tool for optimally predicting survival outcomes and facilitating decision making.

The results of studies concerning the ability of preoperative or perioperative nutritional supplementation to improve postoperative outcomes are controversial. European guidelines for surgical patients support the use of preoperative nutritional supplementation in severely malnourished patients, even if it delays surgery (58). Moreover, a previous study demonstrated that preoperative oral arginine and n-3 fatty acid supplementation improved the immunometabolic host response and outcomes after surgery for CRC (59), while a more recent study proved that protein intake after colorectal surgery was associated with reduced length of hospital stay (60). However, some randomized control trials failed to demonstrate any postoperative benefits from perioperative nutrient therapy, including oral omega-3 fatty acids, eicosapentaenoic acid, or docosahexaenoic acid intake, in patients with CRC surgery (61, 62). Whether or not perioperative nutritional intervention can improve a patient’s long-term outcome thus remains unclear, and further studies are still required to clarify this issue.

The strength of this study was identifying the prognostic role of inflammatory and nutritional status in predicting survival based on standard laboratory factors that are routinely applied in clinical practice. However, the study had some limitations. First, selection bias may have occurred because of the retrospective, single-center nature of the study, and the main findings therefore need external validation in prospective multicenter studies with larger populations. Second, we were not able to include some important confounding factors, including *KRAS*, *BRAF*, or microsatellite instability. Third, the examined lymph nodes in some patients in this study did not meet the minimum 12 lymph nodes requirements of the guidelines, which may have affected the TNM staging. Further verification is therefore required. Finally, although the difference between the novel prediction model and the AJCC 8th TNM classification of CRC was statistically significant, the absolute AUC values were still not qualified for clinical recommendations. More studies are therefore required to validate the main findings of the current study.

## Conclusion

In summary, preoperative nutritional and inflammatory factors have significant prognostic values in patients with CRC. A novel prognostic model incorporating both preoperative inflammatory and nutritional markers provided better prognostic performance than the AJCC 8th TNM classification. The novel developed nomogram incorporating preoperative inflammatory and nutritional markers could individually predict OS and DFS in patients with CRC. These results suggested the need to raise awareness of the importance of preoperative inflammatory and nutritional statuses in patients with CRC. However, the main findings of current study need to be interpreted with caution and require external validation in future studies.

## Data Availability Statement

The raw data supporting the conclusions of this article will be made available by the authors, without undue reservation.

## Ethics Statement

The studies involving human participants were reviewed and approved by Ethics Committee of the Cancer Hospital of China Medical University, Liaoning Cancer Hospital and Institute. The patients/participants provided their written informed consent to participate in this study.

## Author Contributions

Study concept and design: NZ, FN, RG, and CZ. Acquisition of data: NZ, FN, RG, RZ, and CZ. Analysis and interpretation of data: NZ, FN, RG, RZ, and CZ. Drafting of the manuscript: NZ, FN, RG, JP, YQ, JF, BJ, YL, ZC, ZM, MA, JZ, RZ, and CZ. Critical revision of the manuscript for important intellectual content: NZ, JZ, RZ, and CZ. Obtained funding: CZ and ZM. Lead corresponding author: CZ. All authors contributed to the article and approved the submitted version.

## Funding

This work was supported, in part, by the China Scholarship Council (201908050148) and National Natural Science Foundation of China (81774112).

## Conflict of Interest

The authors declare that the research was conducted in the absence of any commercial or financial relationships that could be construed as a potential conflict of interest.

The reviewer NP declared a shared affiliation with several of the authors JF, JZ, to the handling editor at the time of review.
